# Engineering Graphene Phototransistors for High Dynamic Range Applications

**DOI:** 10.1021/acsnano.3c11856

**Published:** 2024-05-10

**Authors:** Shadi Nashashibi, Stefan M. Koepfli, Raphael Schwanninger, Michael Baumann, Michael Doderer, Dominik Bisang, Yuriy Fedoryshyn, Juerg Leuthold

**Affiliations:** ETH Zurich, Institute of Electromagnetic Fields, Zurich 8092, Switzerland

**Keywords:** adaptability, air stable, bioinspired, enhancement, graphene, high dynamic range, phototransistor

## Abstract

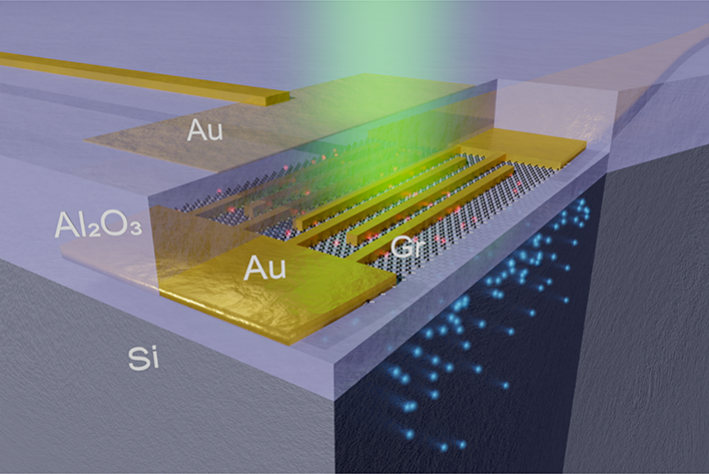

Phototransistors are light-sensitive devices featuring a high dynamic range, low-light detection, and mechanisms to adapt to different ambient light conditions. These features are of interest for bioinspired applications such as artificial and restored vision. In this work, we report on a graphene-based phototransistor exploiting the photogating effect that features picowatt- to microwatt-level photodetection, a dynamic range covering six orders of magnitude from 7 to 10^7^ lux, and a responsivity of up to 4.7 × 10^3^ A/W. The proposed device offers the highest dynamic range and lowest optical power detected compared to the state of the art in interfacial photogating and further operates air stably. These results have been achieved by a combination of multiple developments. For example, by optimizing the geometry of our devices with respect to the graphene channel aspect ratio and by introducing a semitransparent top-gate electrode, we report a factor 20–30 improvement in responsivity over unoptimized reference devices. Furthermore, we use a built-in dynamic range compression based on a partial logarithmic optical power dependence in combination with control of responsivity. These features enable adaptation to changing lighting conditions and support high dynamic range operation, similar to what is known in human visual perception. The enhanced performance of our devices therefore holds potential for bioinspired applications, such as retinal implants.

The advancement of low-dimensional materials has spurred the development of various optical applications including biosensors,^1^ bioimaging,^2^ and photodetectors.^3−5^ The advancements in photodetectors, among others, are driven by the desire to reproduce and improve the capabilities of the human sensory system. Such photodetection devices have moved beyond their traditional role of linearly measuring light intensity and are implemented in a wide range of applications, from neural networks and neuromorphic photodetection^6,7^ to artificial vision systems^8,9^ and vision restoration.^10,11^ In the context of vision-related applications, it becomes imperative to consider the characteristics of the human visual system. Throughout the transition from dawn to dusk, typical retinal illuminance levels encompass a range from 1 to 10^5^ lx.^12^ Human vision effectively covers this extensive range under photopic (day vision) conditions, employing dynamic range compression with an instantaneous intrascene dynamic range of 5 orders of magnitude^12^ at operation speeds of at most 500 Hz.^13^

Two-dimensional (2D) materials have attracted a lot of attention for the realization of photodetectors due to their extraordinary properties. These atomically thin materials with unique electronic and optical properties offer an excellent opportunity to explore photodetection mechanisms and thus advance the field of photodetectors.^14^ An amazing range of capabilities have already been demonstrated with these materials, making them an ideal platform for exploring the next generation of photodetectors to meet the needs of modern sensing applications. Among these demonstrations are photodetectors with mechanical flexibility,^10^ adaptation to changing lighting conditions,^15,16^ neuromorphic features,^17,18^ and high dynamic range photodetection.^19^

Within the realm of two-dimensional photodetectors, phototransistors based on the photogating concept^20−23^ have gained acceptance in terms of low-light detection, high responsivity, and neuromorphic features. Unlike standard photodiodes, which typically accumulate at most one photocarrier per absorbed photon, photogating devices have an inherent gain mechanism. This inherent gain mechanism is often accompanied by a nonlinear power dependence, which in many cases is undesirable. The development of such phototransistors has led to exceptional performance, achieving very high responsivity values of up to 10^10^ A/W^24^ and uncooled detectivity values of up to 10^14^ Jones.^25^ However, these high responsivity and detectivity values are possible because of extended lifetimes of light-generated carriers, reaching up to several seconds. This leads to a trade-off between gain and operation speed. Yet, the discovery of the interfacial photogating effect has introduced a path to fast photogating detection with a fast photoresponse time on the order of 400 ns at the cost of a reduced responsivity on the order of 10^3^ A/W.^26^

Photodetectors based on interfacial photogating have been realized in a number of configurations, revealing the versatility of this photodetection approach. Different channel materials have been introduced, providing a broad study of different channel mobilities and conductivities on photodetection efficiency.^27−29^ The selection of different substrate materials with different band gaps has further demonstrated the potential to extend the spectral sensitivity of the interfacial photogating mechanism.^30,31^ Responsivity values of up to 1.4 *×* 10^4^ A/W have been reported for this type of photogating devices.^32^ The largest reported dynamic range spans five orders of magnitude with a lowest detectable optical power around a nanowatt at responsivities of up to 10^3^ A/W.^26^ This however does not suffice the requirements for vision applications under full daylight conditions. Furthermore, amidst these wide-ranging explorations on interfacial photogating devices, a comprehensive investigation on how the performance is influenced by the graphene channel shape, dimensions, and doping is still lacking. While the use of interdigitated finger structures has been demonstrated for enhanced photocarrier generation and collection and reduction of the contact resistance in 2D-material-based photodetectors,^33−35^ a study on how the dimensions of interdigitated finger structures influences photodetectors based on the photogating mechanism is still missing. Furthermore, double-gate structures have been employed in various photogating-based 2D-material photodetectors for controlling charge carrier distribution;^25,36,37^ however, a study on the advantages of electrostatic doping for performance enhancements in interfacial photogating-based devices remains absent. Finally, a discussion on the potential of nonlinear power dependence and on its advantages for high dynamic range applications is still lacking.

In this work, we demonstrate a graphene-based device utilizing the interfacial photogating effect with picowatt-level photodetection for the visible-wavelength range, covering an illuminance dynamic range from 7 to 10^7^ lx. The high dynamic range operation surpassing the typical retinal illuminance range from dawn to dusk is enabled by a partial logarithmic power dependence closely mimicking human visual perception. Moreover, our photodetector offers the possibility of carefully refining the responsivity, which in turn offers the possibility for adaptive photodetection. Optimizing the geometry of the devices and the addition of a semitransparent top-gate provides a 20- to 30-fold improvement in responsivity compared to not enhanced devices. The geometry of our device, based on interdigitated finger contacts, is optimized with respect to the graphene channel aspect ratio, which can be used to improve the photodetection performance. Additionally, the use of the semitransparent top-gate offers more degrees of freedom to tune the device performance to its ideal operating point. The presented photodetector reliably operates in ambient conditions due to graphene passivation, while the utilization of graphene grown by chemical vapor deposition (CVD), with potential for wafer-scale applications,^38−40^ simplifies the fabrication process and reduces upscaling complexity compared to exfoliated graphene.

## Results and Discussion

### The Proposed Device and Operation Principle

**Figure 1:**
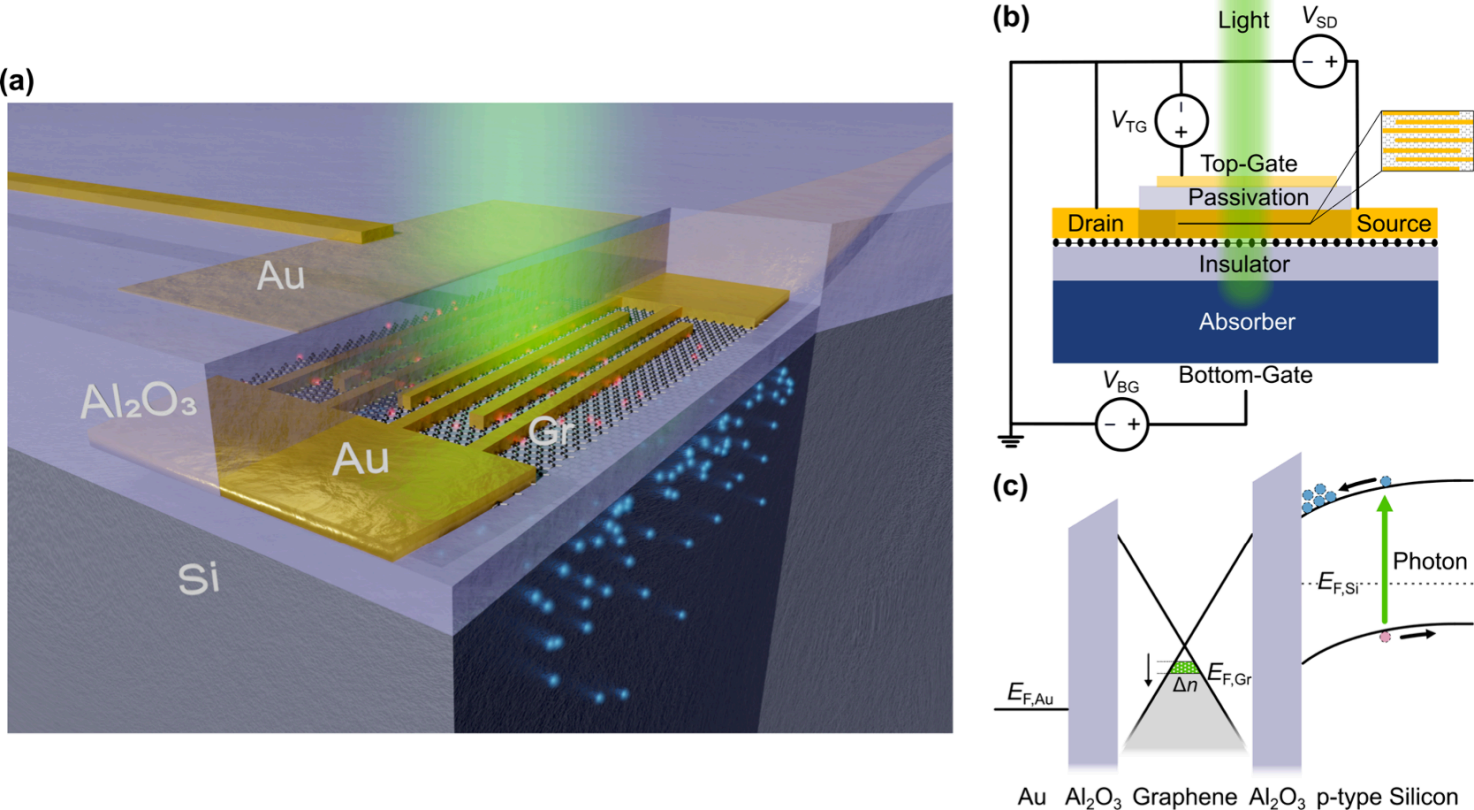
Basic working principles of the proposed graphene-based photodetector. (a) Visualization of the enhanced photogating device. The device consists of an absorbing silicon substrate (Si), an insulating alumina gate oxide (Al_2_O_3_), a graphene patch (Gr), interdigitated source/drain gold finger contacts (Au), an alumina passivation layer (Al_2_O_3_), and a semitransparent gold top-gate (Au). Photogenerated electrons (blue) in the substrate lead to image charges in the graphene patch (red), which affect the conductivity of the graphene patch. (b) Biasing scheme for the enhanced photogating detector. The three voltages *V*_SD_, *V*_TG_, and *V*_BG_ are the source-drain voltage, top-gate voltage, and bottom-gate voltage, respectively. (c) Exemplary band diagram of the device structure (vertical cut) showcasing the absorption of photons, the movement of electrons (blue) and holes (red) in the Si absorber layer, and the photogating effect Δ*n* on the graphene patch.

To investigate the operation of interfacial photogating devices for the visible spectrum, we propose a device as artistically shown in Figure 1a. The interfacial photogating device consists of an absorbing p-type silicon (Si) substrate with a resistivity of 1–10 Ω·cm covered by a 20 nm-thick alumina (Al_2_O_3_) insulating gate oxide layer. On top of the insulating oxide, the CVD-grown monolayer graphene film is transferred and subsequent patterning provides conductive graphene patches. These conductive graphene patches are contacted with gold source-drain contacts from the top, which are shaped in the manner of an interdigitated finger structure, forming a graphene channel. An Al_2_O_3_ passivation layer offers both stability for operation under ambient conditions as well as an insulation between graphene and the 10 nm-thick semitransparent gold top-gate with a transparency of 66% at 532 nm wavelength. The schematic in Figure 1b displays the biasing scheme of the presented device. The source-drain voltage *V*_SD_ is applied to the graphene channel and offers a way to measure the graphene channel resistance. The bottom-gate voltage *V*_BG_ is applied to the bottom of the Si substrate and thus controls both the electronic band bending at the Si/Al_2_O_3_ interface and the Fermi level of the graphene channel. Finally, the top-gate voltage *V*_TG_ is applied to the semitransparent gold film and provides further means to electrically gate the graphene channel and tune the Fermi level thereof.

To clarify the inner working of the photodetector, Figure 1c showcases the band diagram along a vertical cut through the device. Light incident from the top of the device passes through the semitransparent top-gate, passivation, graphene film, and gate oxide and is finally absorbed in the p-type silicon substrate creating photogenerated electron–hole pairs. These photoexcited electron–hole pairs are separated due to the *V*_BG_-controlled band bending of the valence and conduction band of Si. Electrons, being the minority charges in p-type Si, move under the correct biasing conditions toward the insulating Al_2_O_3_ and modify the electrostatic environment of graphene. This shifts the Fermi level in the graphene channel. The subtle change in Fermi level leads to a change in the channel resistance, which then is sensed by applying a source-drain voltage *V*_SD_. The change in source-drain current is the photocurrent *I*_ph_ and can be summarized with the following governing equation:
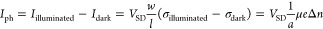
1

Here, σ is the conductivity, *w* is the graphene channel width, *l* is the graphene channel length, *μ* is the mobility of charge carriers in the conductive channel, *e* is the elementary charge, *a* = *l*/*w* is the channel aspect ratio, and Δ*n* is the optically induced change in charge carrier concentration in the graphene channel due to the photogating effect. The photocurrent strength is therefore connected to the material properties by carrier mobility, to the applied bias voltage, the carrier concentration change, and the channel aspect ratio. By having control over Δ*n* through the introduction of the semitransparent top-gate and by varying *a* = *l*/*w* through the use of an interdigitated finger structure, one has the potential to optimize the device response.

The following sections outline the main components of the device. First, the basic operation principles and drawbacks of a standard device without any top-gate or interdigitated finger structure are discussed. By including a semitransparent top-gate, the device can be operated at the optimal operation point improving the photodetection performance. Furthermore, with the help of interdigitated finger structures, one gains control over the aspect ratio of the channel without sacrificing photodetector area and can thus boost the responsivity. Finally, the key photodetection metrics such as responsivity, dynamic range, and operation speed are presented.

### Bottom-Gate Control of the Photodetector

**Figure 2:**
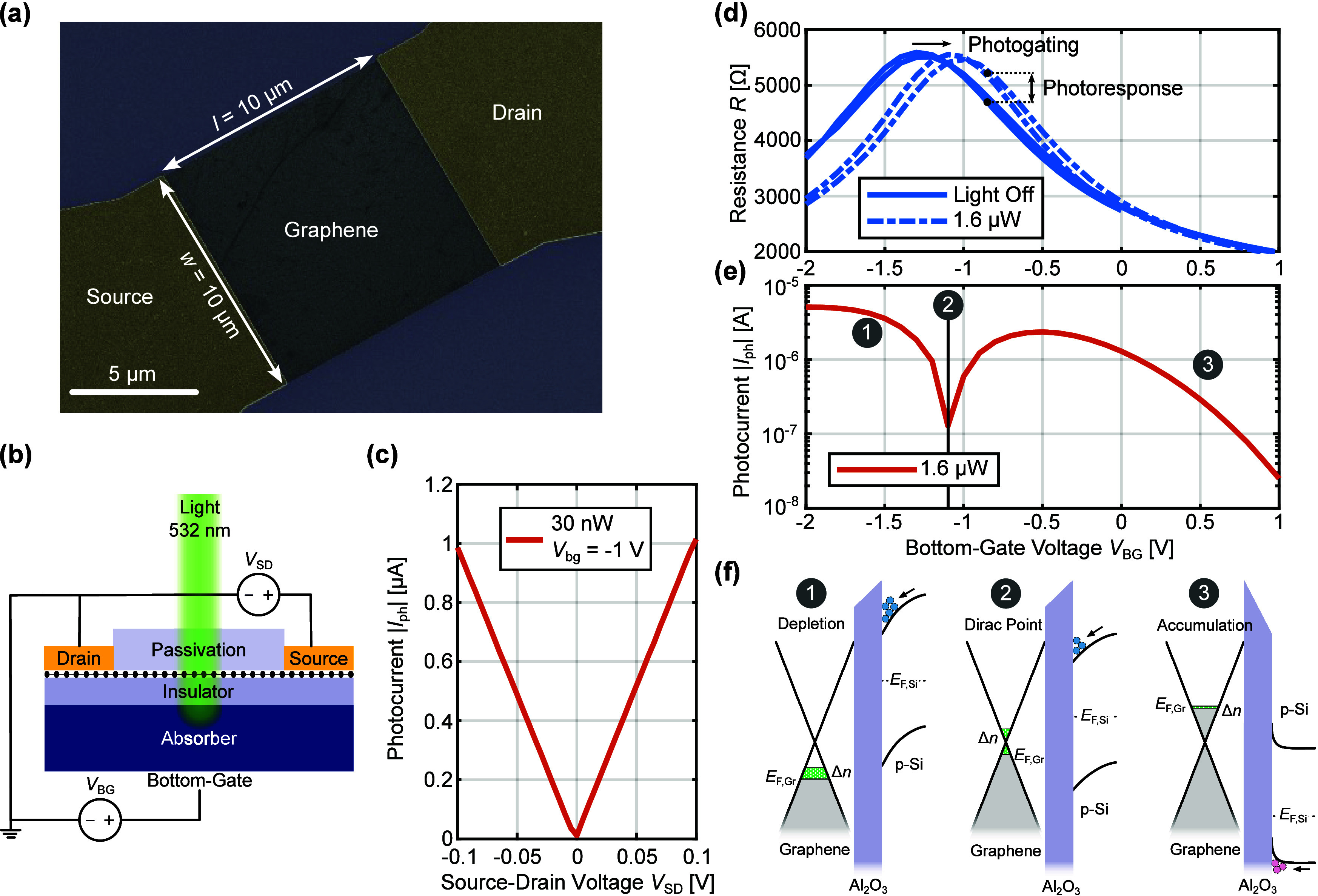
Bottom-gate control of standard interfacial photogating devices under 532 nm illumination. (a) False-colored scanning electron microscopy (SEM) image of a standard device without interdigitated finger contacts or a semitransparent top-gate. (b) Biasing schematic of the standard device with two bias voltages, namely, the source-drain voltage *V*_SD_ and the bottom-gate voltage *V*_BG_. (c) Photocurrent magnitude measured under a source-drain voltage sweep. This behavior nicely follows the expected trend with *V*_SD_ given by eq 1. (d) Channel resistance measured under a bidirectional bottom-gate voltage sweep with and without optical input. Under illumination, the gating curve moves toward the right due to the photogating effect. (e) Bottom-gate voltage dependence of the photocurrent for the device measured in (d) with a source-drain voltage *V*_SD_ of 0.1 V. (f) Band diagrams shown for three distinct bottom-gate voltage conditions. Case 1: The highest response is obtained for the condition with depletion mode biasing of the Si substrate. Case 2: The dip in photocurrent occurs due to the presence of the Dirac point. Case 3: Under positive bottom-gate voltages, the silicon substrate is driven into accumulation leading to a diminishing photoresponse.

To further explore the photodetection mechanism and its dependence on the control voltages *V*_SD_ and *V*_BG_, a device without enhancing features is examined, denoted as a standard device. In Figure 2a, a false-colored SEM image of a device without interdigitated finger structure or semitransparent top-gate is shown. The device consists of a square 10 × 10 μm graphene channel, which is contacted with source and drain contacts, and the absorbing Si substrate acts as a bottom-gate (see Figure 2b), providing two tuning voltages *V*_BG_ and *V*_SD_.

We first test the device’s dependence on the source-drain voltage *V*_SD_, which follows a linear behavior as shown in Figure 2c following the prediction of eq 1. For this purpose, we illuminate the device with a 532 nm laser at 30 nW modulated at a frequency of 525 Hz, while setting a constant gate voltage of *V*_BG_ = −1 V. The measurement isperformed with a lock-in amplifier connected in series to a source-measure unit (SMU) and the device under test (see SI Figure S3 for a schematic of the measurement setup). The electrical signal from the device originating from the modulated laser is extracted, and its amplitude is measured providing a direct and sensitive measure for the photocurrent.

Next, we test the device’s dependence on the bottom-gate voltage *V*_BG_, which is more intricate due to its dual role as an optically and electrically controlled gate, as shown in Figure 2d. The graphene gating curves shown in the figure are obtained by a bidirectional *V*_BG_ sweep with and without the presence of a light source (focused laser spot with 532 nm wavelength). The benefit of the graphene passivation can be clearly observed by the minimally present hysteresis of the gating curves and the stability under ambient conditions (see also SI Figure S4 for a comparison between passivated and nonpassivated devices and SI Figure S13 for photocurrent stability measurements).^35,41^ Compared to the device under dark conditions, the illuminated device with a 532 nm laser power of 1.6 μW exhibits a resistance curve shifted toward higher *V*_BG_ levels, effectively photogating the device (labeled black arrow). By taking the difference between the corresponding currents with and without illumination we obtain the photoresponse (labeled black arrow). The photocurrent shown in Figure 2e is measured with the help of the LIA measurement setup mentioned above.

In order to understand the increased photocurrent under negative *V*_BG_, we must consider the inner working of the device under different *V*_BG_. The photocurrent is higher for negative bottom-gate voltages, where the p-type Si substrate is biased into depletion (see case 1 in Figure 2f and SI Figure S5 for a comparison between n- and p-type silicon substrates). The electric fields introduced from the band bending under depletion biasing offer an efficient means to separate photogenerated electron–hole pairs in silicon and aid the collection of minority charge carriers at the Si/Al_2_O_3_ interface. The low charge carrier concentration in Si under depletion further enables a higher sensitivity to concentration modulations due to collected photogenerated charges. Moreover, the lifetime of minority carriers is prolonged compared to other biasing conditions (see SI Figure S6 for time traces of the photocurrent under different biasing conditions), which leads to a prolonged effect of collected photocharges on graphene channel conductivity allowing charge carriers in graphene to recirculate the channel multiple times. This increases the photogating gain *G* according to the equation given by *G* = *τ*_lt_/*τ*_tr_.^42^ Here, *τ*_lt_ is the minority charge lifetime and *τ*_tr_ the transit time for electrons traveling in graphene from the source to the drain contact. One further intricate feature in the photocurrent curve is the dip close to a bottom-gate voltage at −1 V. This dip is around the Dirac point voltage, where the gating curve flattens out, case 2 in Figure 2f. Thus, an optical modulation in this operation point will lead to a small change in photocurrent and at some voltage the illuminated and nonilluminated gating curves will cross, which ultimately leads to a vanishing photocurrent. One further observation is the decreased photocurrent under accumulation mode biasing for positive voltages, where the electric fields predominantly drop off in the gate oxide (case 3 in Figure 2f). The high charge carrier concentration at the Si/Al_2_O_3_ interface biased under accumulation mode further leads to a diminishing sensitivity to concentration modulations. The same argument follows for the case of inversion mode biasing under large negative *V*_BG_.

Ideally, the interfacial photogating device is operated at a point where the photogating effect is most pronounced. This requires two simultaneous conditions: (1) the device is operated at a point where the maximal collection of minority charge carriers in silicon is achieved and (2) where these charges have the most pronounced effect on the graphene channel’s conductivity. While the most efficient collection is obtained under depletion mode biasing of the bottom-gate (see Figure 2e,f), the Dirac point voltage with respect to *V*_BG_ is given by uncontrollable residual dopants on graphene after fabrication and leads to a dip in the photocurrent. The electronic and photoelectric effects are both controlled by the bottom-gate voltage for devices equipped solely with a bottom-gate. Therefore, there is no full control over the operation point of the device. The next section discusses how the top-gate allows to gain full control over the device operation point.

### Introducing the Semitransparent Top-Gate Structure

**Figure 3:**
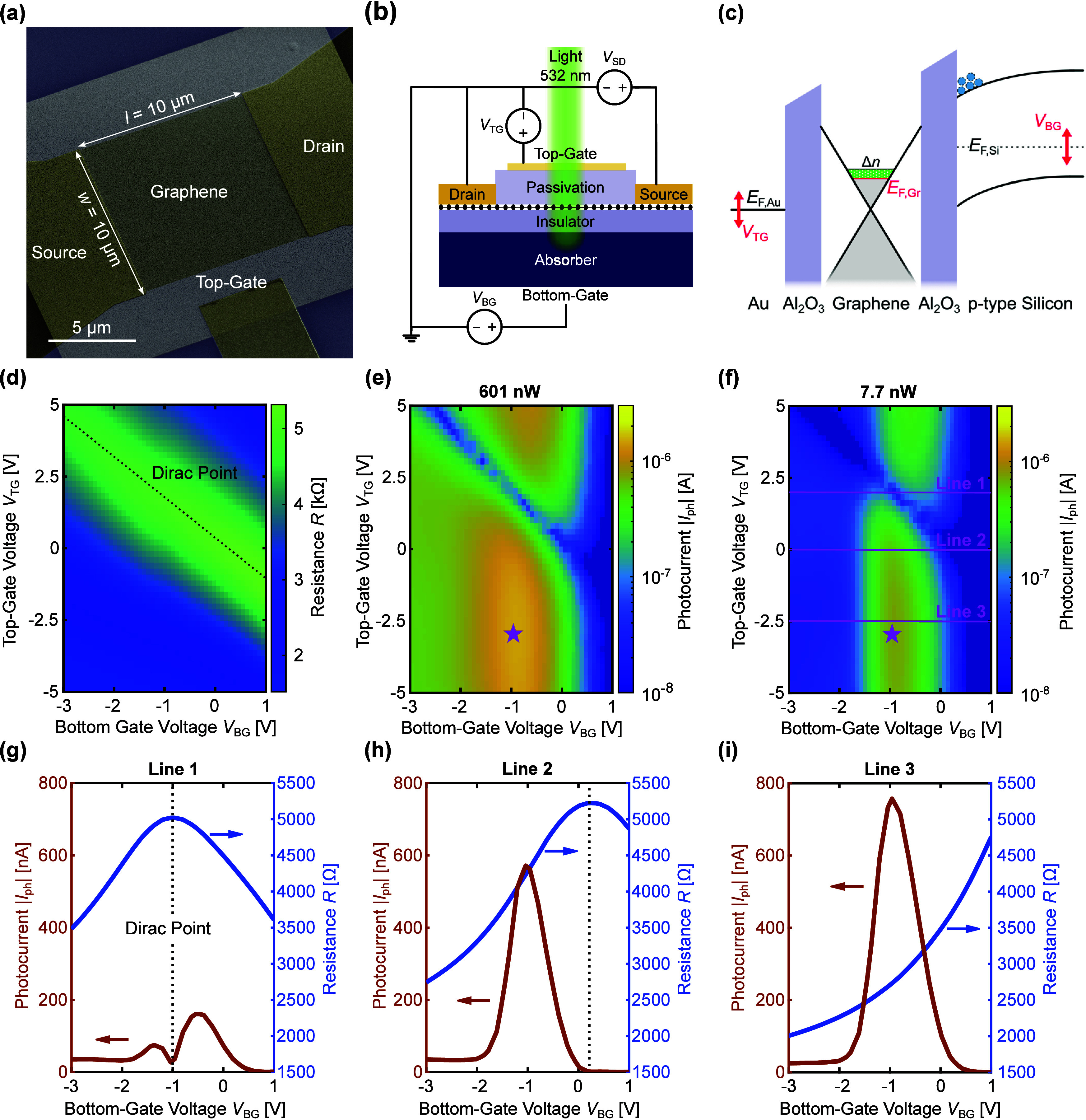
Performance of interfacial photogating devices with a semitransparent top-gate under 532 nm illumination and a source-drain voltage *V*_SD_ of 0.1 V. (a) False-colored SEM image showing an interfacial graphene photogating device with a semitransparent top-gate. (b) Biasing schematic of the device with three control voltages, namely, the bottom-gate voltage *V*_BG_, the source-drain voltage *V*_SD_, and the top-gate voltage *V*_TG_. (c) Band diagram for a device with a semitransparent top-gate. The bottom- and top-gate voltages control the Fermi level in the absorbing Si substrate *E*_F,Si_ and the top-gate *E*_F,Au_, respectively, and thus tune the graphene Fermi level *E*_F,Gr_. (d) Graphene channel resistance measured in dependence of the bottom-gate and top-gate voltage under dark conditions. (e, f) Photoresponse in dependence of the bottom- and top-gate voltages for 601 and 7.7 nW, respectively, of optical power. The maximum photocurrent is marked with a magenta-colored star. (g–i) The photocurrent (left axis) for the three horizontal labeled lines highlighted in magenta in (f) are shown in more detail together with the gating resistance curve (right axis). The location of the Dirac point (black dotted line) has a detrimental effect on the highest achievable photoresponse.

To achieve ideal operation conditions, we introduce the semitransparent top-gate to the interfacial photogating device with which the Dirac point can be freely shifted with respect to *V*_BG_. This allows for the correction of differences in graphene doping, which is difficult to control and introduced by the graphene transfer process. Figure 3a shows an SEM image of a device with a semitransparent top-gate. The top-gate consists of a 10 nm thin gold film grown on top of the 50 nm thick passivation layer and offers a transparency of 66% at the laser wavelength of 532 nm. Aside of the source-drain voltage *V*_SD_ and the bottom-gate voltage *V*_BG_, the newly introduced top-gate voltage *V*_TG_ enables gating of the graphene channel from the top (outlined in Figure 3b,c). This is shown in Figure 3d, where a measured two-dimensional map of the resistance in dependence of *V*_BG_ and *V*_TG_ is plotted. The top-gate acting as a purely electronic gate is capacitively coupled to the graphene channel and thus allows for additional control over the conductivity. We therefore can decouple the effects from the photogate from the electrostatic graphene doping and tune the gating curve of graphene to a position of highest sensitivity to changes in the photogate.

The ideal operation point is independent of the optical input power, as can be seen from photocurrent measurements shown in Figure 3e,f. The maximal photocurrent is indicated by a magenta-colored star and is achieved for a bottom-gate voltage and top-gate voltage of around −1 and −2.5 V, respectively. A bottom-gate voltage of approximately −1 V leads to most pronounced photocurrents regardless of *V*_TG_ showcasing the importance of biasing the silicon into depletion mode. For the measurement of these two-dimensional heat maps, both the bottom- and top-gate voltages are swept and the photocurrent is measured with a LIA for the device shown in Figure 3a under laser illumination with 601 and 7.7 nW at a modulation frequency of 525 Hz.

By introducing the top-gate, we control the graphene channel resistance independently from the bottom-gate voltage, which is essential to tune the device into the best-performing point by optimizing Δ*n*. To highlight these benefits of using the top-gate, we further compare three specific operation points of the top-gate in the heat map, as presented with magenta-colored lines in Figure 3f. The photocurrent (left axis) and resistance (right axis) in dependence of the bottom-gate voltage for the first cutline (“Line 1”) at a top-gate voltage of 2 V are shown in Figure 3g. The Dirac point with respect to the bottom-gate *V*_BG_ lies at a voltage of around −1 V. As discussed above (case 2 in Figure 2f), the operation at the Dirac point leads to an adverse effect in the achievable photoresponse. Thus, the graphene Dirac point position interferes with the ideal *V*_BG_ operation in depletion mode. Δ*n* in this case is minimized although the charge carrier collection at the Si/Al_2_O_3_ interface is at its most efficient point. Under top-gate voltages of 0 and −2.5 V as shown in Figure 3h and i, respectively, the Dirac point is driven sufficiently far away from the ideal bottom-gate voltage operating point and thus the photocurrent reaches higher values. As the doping concentration is not controlled during the fabrication processes, the device could have an arbitrarily positioned intrinsic Dirac point position. Thus, by controlling the graphene doping with the top-gate, we can improve the performance for the worst-case scenario as shown in Figure 3g by up to 4.7× as shown in Figure 3i. Within the framework of eq 1, we have successfully optimized the optically induced charge carrier shift Δ*n*. We next target the effect of the graphene channel’s aspect ratio *a* = *l*/*w* and its effects on the photocurrent.

### The Interdigitated Source-Drain Finger Structure

**Figure 4:**
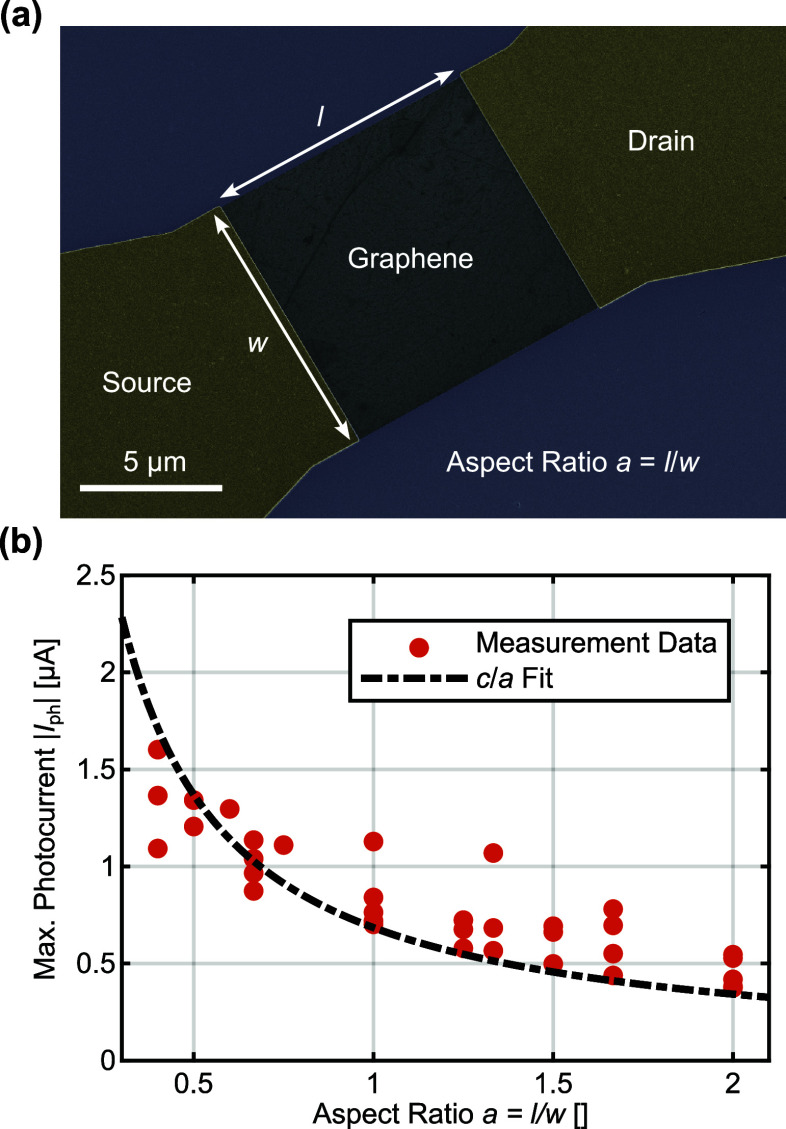
Performance of interfacial photogating devices with rectangularly shaped channels under 532 nm illumination and a source-drain voltage *V*_SD_ of 0.1 V. (a) False-colored SEM image of a standard interfacial photogating device with a rectangularly shaped graphene channel with width *w* and length *l*. The aspect ratio *a* of the channel is defined as *l*/*w*. (b) Maximal photocurrent in dependence of the channel aspect ratio. The black curve shows a numerical fit obtained with the equation *I*_ph_ = *c*/*a*, where *c* is a proportionality constant.

According to eq 1, the photocurrent increases with width and decreases with length of the graphene channel. We have measured the maximal photocurrent for a total of 34 devices with varying aspect ratios experimentally verifying these dependencies. Figure 4a shows an SEM image of an exemplary standard device with a rectangularly shaped graphene channel, where the fabrication of the semitransparent top-gate has been omitted. Every device is measured with a *V*_BG_ optimized for the highest photocurrent acting as a standardized metric for comparing devices by mitigating the effect of nonuniform graphene quality. Figure 4b shows the maximum photocurrent in dependence of the aspect ratio of all 34 devices measured. The black dashed curve is a fitting curve, formulated as *c*/*a* as given by proportionalities of eq 1, where *c* represents a proportionality constant and *a* = *l*/*w* denotes the channel’s aspect ratio. The data closely follows the *c*/*a* behavior and shows that decreasing the aspect ratio indeed leads to an increase in photocurrent. Small deviations from the fitted function are present for both large and small aspect ratios. These deviations arise from the fact that for small aspect ratios where the channel is short and wide, a part of the incident light is reflected by the source-drain contacts. Thus, there is less light reaching the absorbing silicon substrate, which leads to a weaker photogating effect. For large aspect ratios, the channel is long and narrow and thus more light reaches the silicon substrate leading to a more pronounced photogating effect.

**Figure 5:**
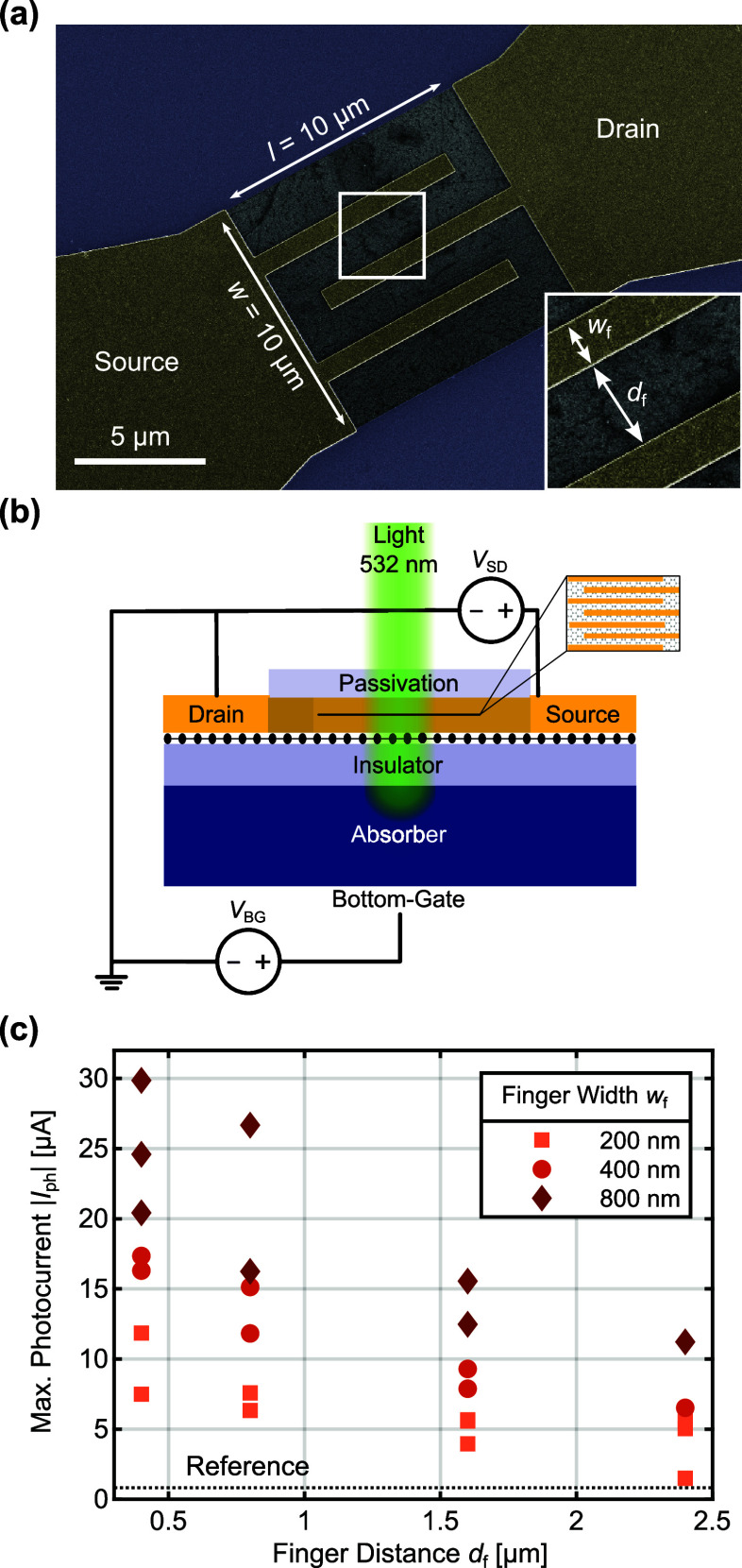
Performance of interfacial photogating devices with interdigitated finger contacts under 532 nm illumination and a source-drain voltage *V*_SD_ of 0.1 V. (a) SEM image of an interfacial photogating device with an interdigitated source-drain finger structure to contact the 10 × 10 μm graphene patch. The fingers have a finger width *w*_f_ and finger distance *d*_f_ as defined in the image. (b) Biasing scheme for a device with interdigitated fingers with source-drain voltage *V*_SD_ and bottom-gate voltage *V*_BG_. (c) Maximal photocurrent for different interdigitated finger configurations. By decreasing the finger distance, the channel aspect ratio is artificially reduced and the photoresponse increases while keeping the photodetector area constant. The dotted reference line denotes the photoresponse for a standard device with a 10 × 10 μm graphene channel.

The interdigitated finger structure for the source-drain contacts mimics a short and wide channel and therefore offers a pathway to artificially decrease the channel aspect ratio without affecting the active device area. Figure 5a shows an SEM image of a 10 × 10 μm-sized device with interdigitated finger contacts. Two new parameters for this device are defined: the finger width *w*_f_ and the finger-to-finger distance *d*_f_. The number of fingers  is given by the maximum amount, which can be fitted into the *w* = 10 μm-wide channel. In a first-order approximation, the channel aspect ratio of the finger structure *a*_f_ can be defined as , where *l* = 10 μm is the graphene channel length. Rather than modifying the graphene patch shape for lower aspect ratios, the aspect ratio of the finger structure can now be reduced solely by optimizing the two parameters *d*_f_ and *w*_f_. This ultimately affects the gain equation *G* = *τ*_lt_/*τ*_tr_ by reducing the transit time *τ*_tr_ with which we obtain higher gain values. Figure 5b illustrates the biasing scheme for devices with interdigitated fingers. Again, the top-gate has been omitted; as such, the devices have only two control voltages, namely, the source-drain voltage *V*_SD_ and the bottom-gate voltage *V*_BG_.

Decreasing the finger distance has a beneficial effect on the photoresponse by creating ever smaller artificial channel aspect ratios *a*_f_. This is displayed in Figure 5c, where the measurement of the maximum photocurrent in dependence of finger distance and width for 25 devices with varying interdigitated finger configurations is shown. It is further found that wider fingers increase the maximum photocurrent. We attribute this to three effects, namely, the reduced contact resistance between the finger contacts and graphene as well as the reduced finger resistance due to wider finger contacts (see SI Figure S7). Furthermore, a larger finger width reduces the number of fingers, which can be fitted into the 10 μm-wide channel and thus the reflection of the interdigitated finger structure is further reduced, which in turn leads to a larger amount of light being absorbed in the silicon substrate. A numerical study on the reflection behavior of the interdigitated finger structure can be found in SI Figure S8.

### High Dynamic Range Operation and Adaptability

**Figure 6:**
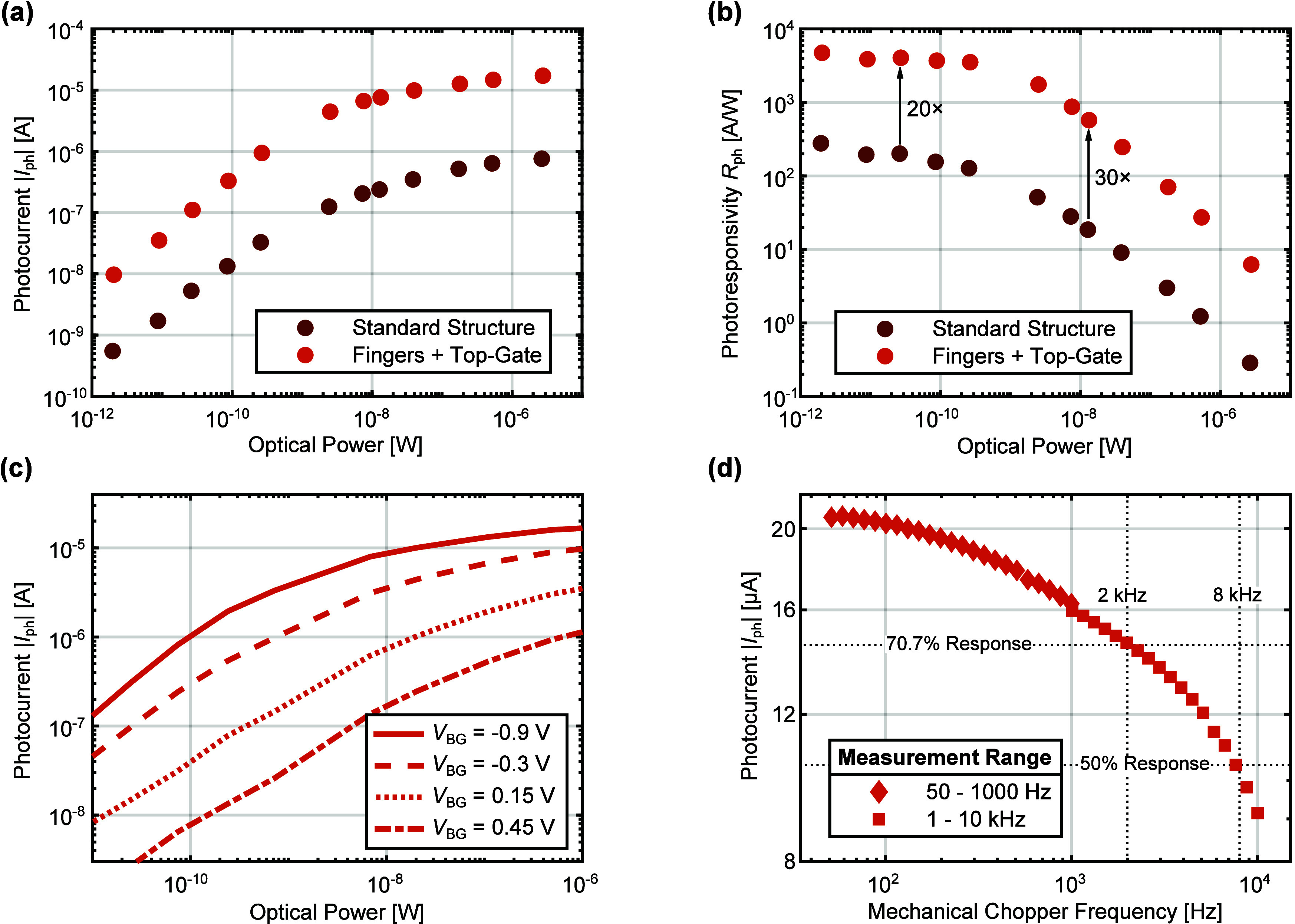
Performance metrics of the enhanced interfacial photogating device under 532 nm illumination and a source-drain voltage *V*_SD_ of 0.1 V. Photocurrent (a) and responsivity (b) for an optical power sweep across six orders of magnitude for a standard and an enhanced device. The standard device has a 10 × 10 μm channel without any enhancements, while the enhanced device incorporates a finger structure (*d*_f_ = 1.2 μm and *w*_f_ = 0.8 μm) and a semitransparent top-gate biased at the optimal operating point (*V*_TG_ = 1.5 V). In both cases, the bottom-gate voltage has been set to −0.9 V providing optimal conditions for photodetection. (c) Power sweep measurements at different bottom-gate voltages shifting the response curve. This displays the ability to tune the photocurrent to the desired range and adapt the responsivity depending on the ambient light conditions. (d) Frequency response of the enhanced device for a mechanically chopped optical input at 532 nm wavelength at the ideal operating point. The two measurement ranges correspond to two different chopper blades used to measure the two modulation ranges from 50 to 1000 Hz and 1–10 kHz.

To fully harness the enhancements of the interdigitated finger structures and the semitransparent top-gate, the envisioned device introduced in Figure 1 has been fabricated and its performance metrics were measured, as summarized in Figure 6. The measured photocurrent and responsivity are shown in Figure 6a and b, respectively, across six orders of magnitude for a standard and enhanced device with an interdigitated finger structure (finger width of 0.8 μm and a finger-to-finger distance of 1.2 μm) and a semitransparent top-gate on top of the passivation. The responsivity is calculated by taking the ratio of the photocurrent and the incident optical power. The dynamic range spans from 2 pW up to several μW, which outperforms the prior art of interfacial photogating devices in terms of low-light detection and dynamic range, to the best of our knowledge (see also SI Figure S10 for a comparison). This high dynamic range is compressed down to roughly three orders of magnitude in photocurrent due to a built-in logarithmic compression. In terms of illuminance, the photodetector covers the range from 7 to 10^7^ lx (calculated under monochromatic conditions; the conversion calculation procedure can be found in the SI Figure S11), which closely matches the typical retinal illuminance levels from dawn to dusk (1 to 10^5^ lx). The enhanced device with the interdigitated fingers and the top-gate performs 20× to 30× better compared to the standard structure under the same operation conditions in terms of responsivity. The specific detectivity of the enhanced device is given by  5.2 × 10^9^ Jones, where *R*_ph_= 4.7 × 10^3^ A/W is the responsivity, *A* = 10^–6^ cm^2^ is the active area, and *S*_n_= 9 × 10^–10^ A/Hz^0.5^ is the measured current spectral noise density. The measurements are performed with a 532 nm laser modulated at 525 Hz and a source-drain voltage of 0.1 V resulting in dark currents of 30 and 478 μA for the standard and enhanced devices, respectively. These dark currents are a result of the lack of a band gap in graphene, which limits the noise performance and leads to an increased power consumption. The optical power sweep measurements is performed at a bottom-gate voltage *V*_BG_ of −0.9 V, where the charge carrier collection in Si is most efficient. Furthermore, the enhanced structure’s top-gate is biased at a top-gate voltage of 1.5 V yielding the best performance of the device. Here the optimal top-gate voltage is different from the values shown in Figure 3 as the graphene has a different intrinsic doping level and thus requires a different top-gate voltage to optimize for the best possible performance. Measurements of the spectral responsivity of an enhanced device in dependence of optical power can be found in SI Figure S9.

Two different power dependence regimes for the photocurrent can be distinguished. For higher power levels above 1 nW, the interfacial photogating effect renders a logarithmic dependence on optical input power. This closely matches the human visual system, where perception depends on the stimuli logarithmically according to the Weber–Fechner law.^43^ For power levels below 1 nW, the dependence of the photocurrent on the optical input power is close to linear. The loss of the logarithmic dynamic range compression at these low optical powers is not fully understood and requires further investigation. Photodetection of optical powers smaller than pW levels has been limited by noise. Compared to the standard structure, the enhanced device with the interdigitated fingers and top-gate performs up to 30× better in the logarithmic regime and 20× in the close-to-linear regime. Due to these intricate power dependencies, the proposed device offers a built-in dynamic range compression suitable for high dynamic range applications. This contrasts with traditional approaches, where the dynamic range compression is performed with circuitry and linear photodiodes.^44^

By changing *V*_BG_, the operation range can be shifted with regard to the optical input power offering a means to adapt the responsivity of the photodetector. In Figure 6c, power sweep measurements for an enhanced device with different bottom-gate voltage conditions are shown. Under low-light conditions, the photodetector can be biased with a bottom-gate voltage of −0.9 V offering the highest responsivity. For bright conditions on the other hand, the bottom-gate voltage can be tuned to, e.g., 0.45 V, where the photodetection mechanism is less sensitive to the input power. This offers a method to fine-tune the gain of the photodetector within a suitable range for prevailing lighting conditions relaxing the requirements of the readout circuitry in terms of noise characteristics and high dynamic range photocurrent handling capability, as is required for traditional image sensors.

In terms of operation speed, the flat frequency response is below 100 Hz and the frequency, where the current response drops to 50%, is 8 kHz as shown in Figure 6d, offering sufficiently fast photodetection for image sensing and artificial vision applications. The frequency sweep measurement of the enhanced device is performed at a bottom-gate voltage of −1 V and a top-gate voltage of 1.5 V at an optical input power of 30 nW. This measurement is carried out with a lock-in amplifier by changing the modulation frequency of the mechanical chopper from 50 Hz to 10 kHz for which two different chopper blades are required. The frequency response of the device is limited by the fall time and is dependent on the bottom-gate voltage and optical input power due to changing minority charge carrier lifetimes, which is outlined in SI Figure S12. Time-trace measurements have been performed as shown in SI Figure S6, for which we extract a fall time of 1.2 ms for a bottom-bate voltage of −1 V.

## Conclusions

The demonstrated phototransistor leverages an enhanced interfacial photogating mechanism employing a passivated and air-stable CVD-graphene channel, which is sensitized by an absorbing silicon substrate. The device features a dynamic range of six orders of magnitude from pW to μW levels (7 lx to 10 Mlx in terms of illuminance). The high dynamic range in combination with the pW level photodetection outperforms state-of-the-art devices based on the interfacial photogating mechanism. Our photodetector further features a built-in dynamic range compression, which is based on a partial logarithmic optical power dependence. The six orders of optical dynamic range are ultimately reduced to photocurrent variation across three orders of magnitude. We have further introduced two enhancement techniques that amplify the photodetection performance by up to a factor of 30. First, by incorporating a semitransparent top-gate, we achieved precise control over the photodetector’s operational point. Second, employing interdigitated finger structures allowed us to artificially adjust and optimize the aspect ratio of the graphene channel, enhancing the photoresponse. Such enhancements are not limited to interfacial photogating detectors but can be adapted for a broad range of photogating devices. To complement the aforementioned features, we have shown an adaptive responsivity control based on bottom-gate voltage control. This feature enables fine-tuning of the responsivity to accommodate for different lighting conditions effectively. This work therefore offers a route to high dynamic range imagers based on graphene phototransistors exploiting the nonlinear power dependence and gain control. These combined capabilities prove invaluable for applications demanding high dynamic range operation, such as artificial and restored vision.

## Experimental Section

### Device Fabrication

The photodetector fabrication started with the plasma-enhanced atomic layer deposition (ALD) of a 20 nm thick Al_2_O_3_ layer on a silicon substrate (1–10 Ω·cm, 525 μm thick). The commercially available CVD-grown monolayer graphene has been transferred using either a wet transfer method with a PMMA support layer or the “Easy Transfer” method from Graphenea. The transferred graphene layer was then patterned and etched using e-beam lithography (EBL) and a reactive ion etching process. The source and drain contacts have been patterned by EBL and deposited by e-beam evaporation (EBE) of Ti/Au (5/60 nm) with a subsequent lift-off process. The subsequent step of graphene passivation was obtained by depositing 50 nm of Al_2_O_3_ with low-temperature thermal ALD. The contact pads were opened by photolithography and phosphoric acid wet etching. Finally, the 10 nm-thick semitransparent gold top-gate has been obtained by EBL, EBE, and a lift-off process. Contacts for the top-gate were obtained by EBL patterning, EBE of Ti/Au (5/100 nm), and a lift-off process.

### Device Characterization

The measurement setup used for device characterization is shown in SI Figure S4. To characterize the photoresponse of the photodetectors, devices were directly illuminated with a laser from the top. The laser was first attenuated with neutral density filters and then mechanically chopped at a desired modulation frequency. The laser beam was then focused with a 10× objective onto the device under test. The source-drain and top-gate contacts were contacted from the top with a DC probe, which was connected in series to a source-measure unit (SMU) and a lock-in amplifier (LIA). The bottom-gate was contacted using a conductive chuck allowing voltages to be applied from the bottom of the silicon substrate with an SMU.

## Data Availability

The data that support the findings of this study are available from the corresponding authors on reasonable request.

## Supporting Information Available

Raman characterization of graphene; graphene mobility characterization; measurement setup for photodetector characterization; importance of the Al_2_O_3_ graphene passivation layer; comparison of n- and p-type silicon; time trace of the photocurrent; resistance of devices with and without interdigitated finger structures; reflection simulations of interdigitated finger structures; spectral responsivity at different optical powers; comparison to demonstrations in literature; photometric calculations; power and bias dependence of frequency measurements; stability measurement of the presented photodetectors; comparison of various photogating devices (PDF)
