# Supplementary material for: Engineering Graphene Phototransistors for High Dynamic Range Applications
Source: ACS Nano. 2024 May 10;18(20):12760–70. doi: 10.1021/acsnano.3c11856 (PMC11112981; doi:10.1021/acsnano.3c11856)
Supplement: Supplementary file 1 — nn3c11856_si_001.pdf [file nn3c11856_si_001.pdf]

Supporting Information

Engineering Graphene Phototransistors for High Dynamic Range Applications

Shadi Nashashibi, Stefan M. Koepfli, Raphael Schwanninger, Michael Baumann, Michael Doderer, Dominik Bisang, Yuriy Fedoryshyn, Juerg Leuthold**

Institute of Electromagnetic Fields, ETH Zurich, 8092 Zurich, Switzerland

Corresponding Author

Shadi Nashashibi - Institute of Electromagnetic Fields, ETH Zurich, 8092 Zurich, Switzerland, Email: nshadi@ethz.ch

Juerg Leuthold - Institute of Electromagnetic Fields, ETH Zurich, 8092 Zurich, Switzerland, Email: leuthold@ethz.ch

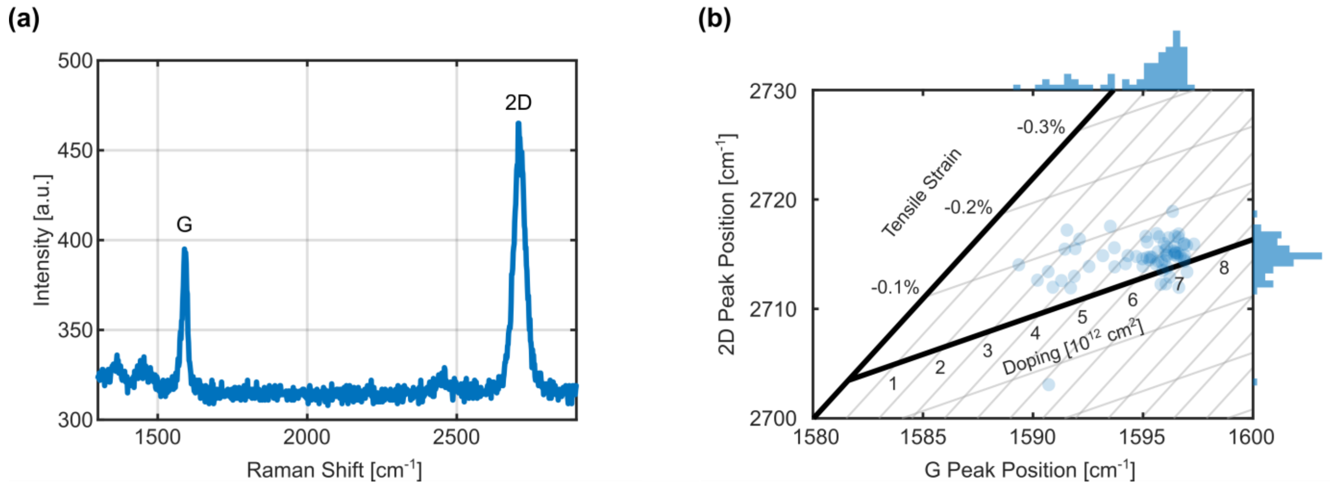

Figure S1. Raman characterization of graphene

(a) Raman spectrum of graphene as used for the proposed device after the transfer process (layer stack consisting of silicon, 20 nm Al_2O_3 and graphene) recorded using a 471 nm laser. (b) A total of 100 Raman spectra as shown in (a) have been measured on an area of 100×100 μm^2. Taking into consideration the position of graphene's G and 2D peak for each measured spectrum (obtained by Lorentzian fitting), one can assemble a two-dimensional plot, where each point corresponds to a Raman measurement on a different location of the graphene sample. This information can be translated into information on graphene's doping and strain levels.[1] The data shows that the transferred graphene has very low strain/stress, and a moderate amount of doping. Based on our observations, it is anticipated that the introduction of a passivation layer results in a reduction in both strain and doping levels.[2]

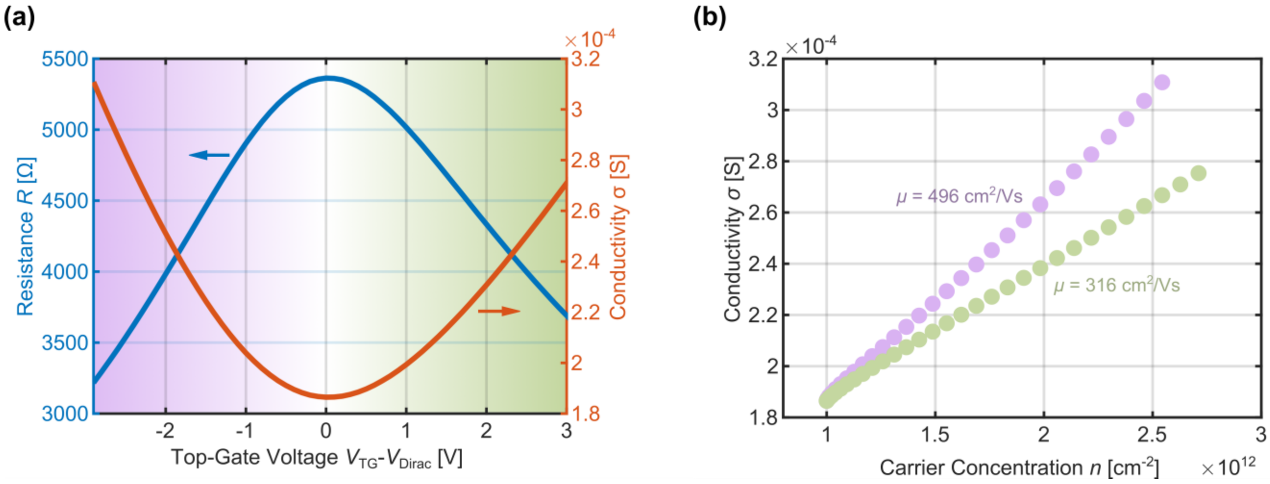

Figure S2. Graphene mobility characterization

(a) Graphene gating curve for a interfacial photogating device obtained by sweeping the top-gate voltage. The resistance (shown in blue, left axis) and the conductivity (shown in red, right axis) are given in dependence of the top-gate voltage shifted by the Dirac point voltage. A differentiation is made between the area left (violet) and right (green) of the Dirac point. (b) Graphene conductivity in dependence of the carrier concentration of graphene calculated according to the procedure shown by Miseikis *et al.*[3] This plot provides a means to extract the mobility of the graphene channel by considering $\mu = \frac{1}{e}\frac{\Delta\sigma}{\Delta n}$. The estimated graphene mobility for the region to the left of Dirac point (violet) is 496 cm^2/Vs and for the region to the right of the Dirac point (green) is 316 cm^2/Vs.

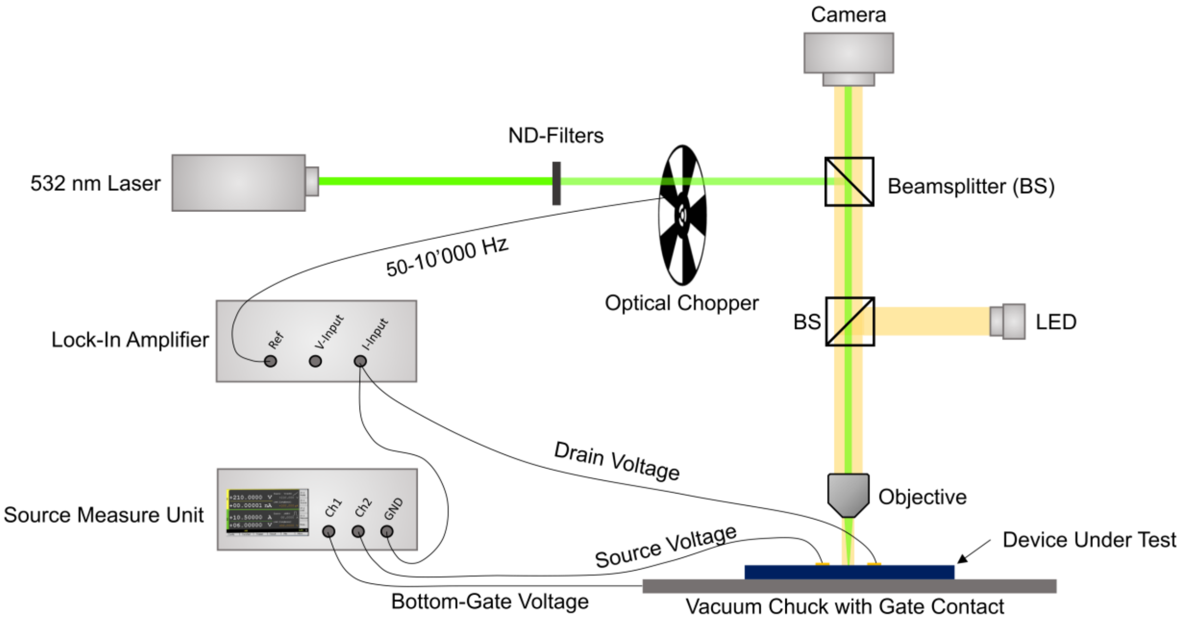

Figure S3. Measurement setup for photodetector characterization

The figure shows the measurement setup as used for the characterization of photogating devices. A 532 nm laser is attenuated with help of neutral density (ND) filters and modulated with an optical chopper at a frequency given by the reference signal of the lock-in amplifier (LIA). The laser beam is directed to the objective of the microscope (5× or 10× magnification). For alignment purposes a white LED is used to illuminate the sample. The objective focuses the incoming light onto the device under test, which is probed with help of DC-needles. The device is connected in series with a source measure unit (SMU) and a LIA. The SMU is used to apply the source-drain bias, the top-gate voltage (if applicable) as well as the bottom-gate voltage.

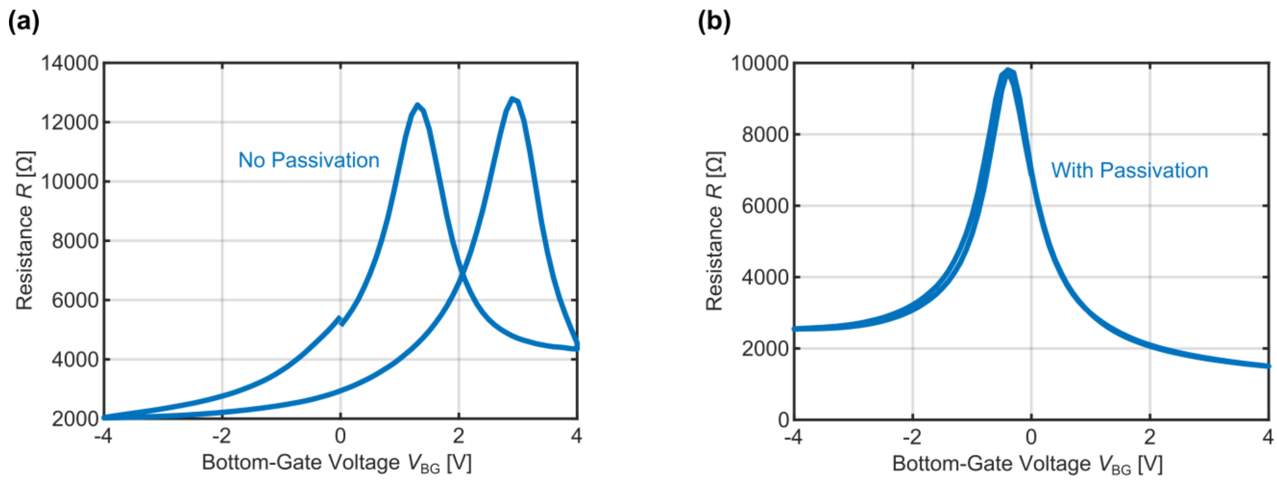

Figure S4. Importance of the Al_2O_3 graphene passivation layer

(a) Graphene gating curve shown as the resistance of a graphene channel in dependence of the bottom-gate voltage. One can clearly observe a large hysteresis suggesting strong atmospheric effects on graphene. (b) Introducing the 50 nm ALD-grown Al_2O_3 graphene passivation layer[2] removes the hysteresis and shifts the Dirac point closer to 0 V, suggesting an improvement in graphene quality. This measurement has been performed on a different chip than the measurement shown in (a).

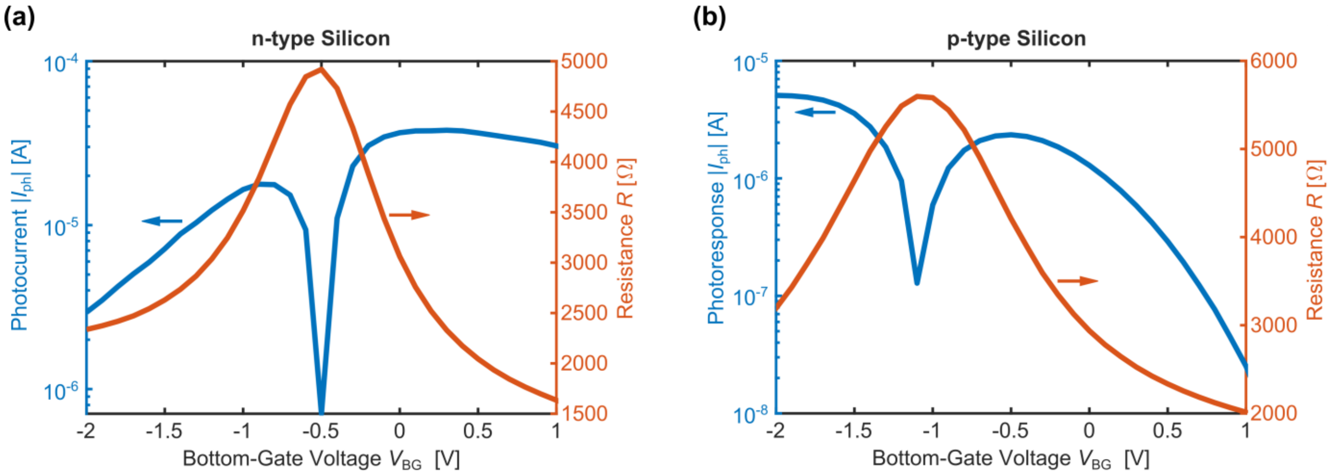

Figure S5. Comparison of n- and p-type silicon

(a) Photocurrent and resistance measurements performed for a standard photogating device with an n-doped silicon substrate in dependence of the bottom-gate voltage. The photocurrent shows the strongest response for positive voltages due to the depletion mode biasing. (b) Comparing the results with a p-type silicon substrate device clearly shows the difference with respect to doping. For a p-type silicon substrate negative bottom-gate voltages must be in order to achieve depletion mode biasing.

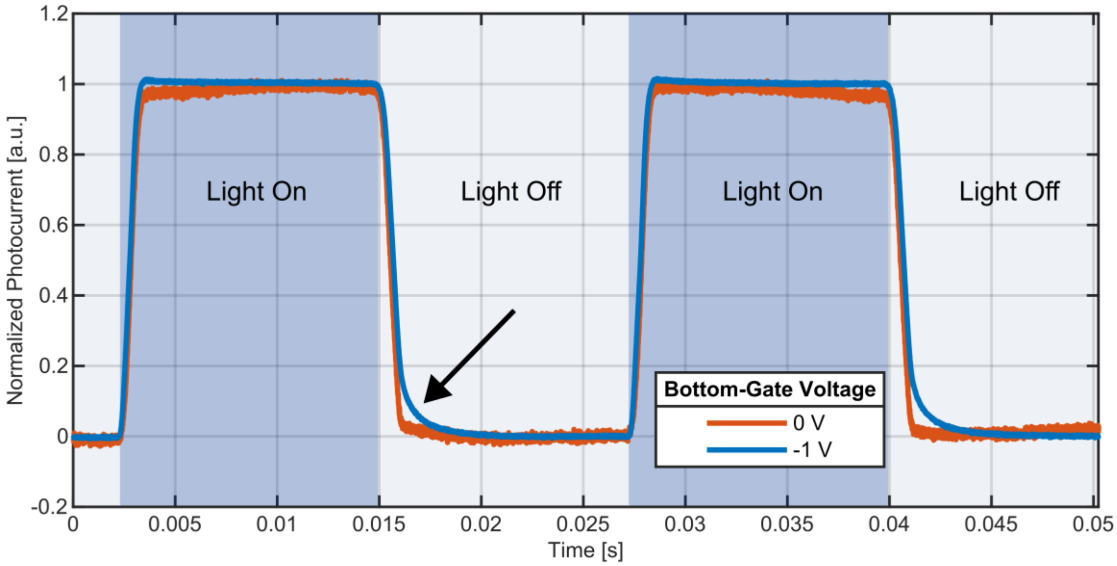

Figure S6. Time trace of the photocurrent

The figure shows normalized photocurrent time trace measurements of the proposed device with a modulated 532 nm laser. The measurements were performed with a source-drain voltage of 0.1 V. Under a bottom-gate voltage of -1 V (blue curve), the silicon substrate is biased into depletion mode resulting in a prolonged lifetime of minority charge carriers in silicon. This is clearly visible by considering the slower recovery of the photocurrent signal indicated with a black arrow as compared to the red curve with a bottom-gate voltage of 0 V (red curve).

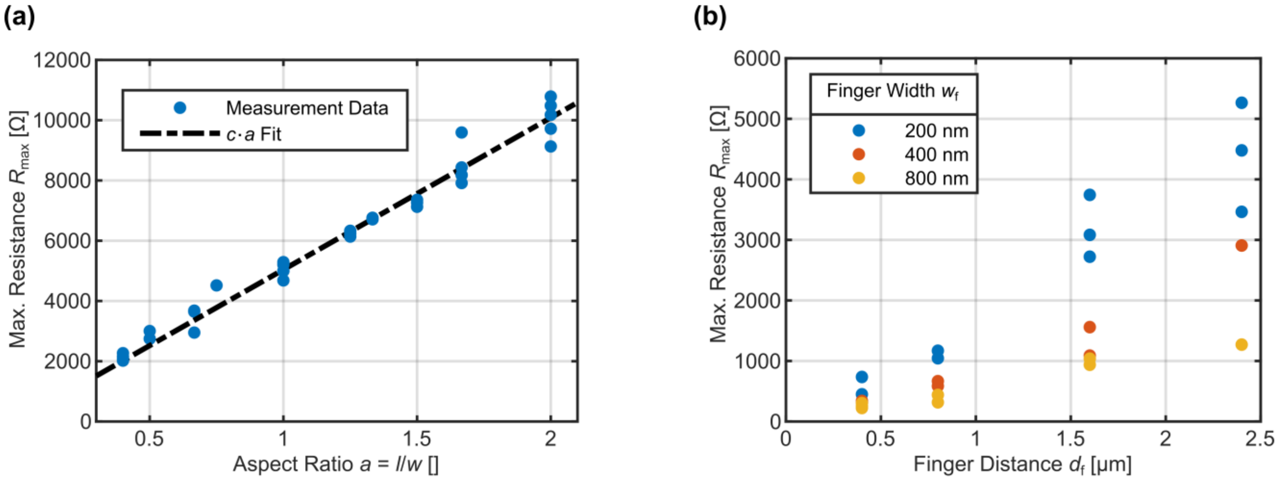

Figure S7. Resistance of devices with and without interdigitated finger structures

(a) Max. resistance (resistance at the Dirac point) measurements of interfacial photogating devices without enhancement in dependence of the aspect ratio $a = l/w$ of the rectangularly shaped graphene channel. The resistance values perfectly follow a linear behaviour as expected by $R = \frac{1}{ne\mu} \cdot a$. The measurement corresponds to the devices shown in Fig. 4 of the main text. (b) Max. resistance measurement of interfacial photogating devices with interdigitated finger structures. The resistance is given in dependence of the finger-to-finger distance d_f and the finger width w_f. One can clearly observe an increase in resistance with increasing finger distance and decreasing finger width. The dependence on finger width is given by an increased finger and contact resistance. The measurements correspond to the devices shown in Fig. 5 of the main text.

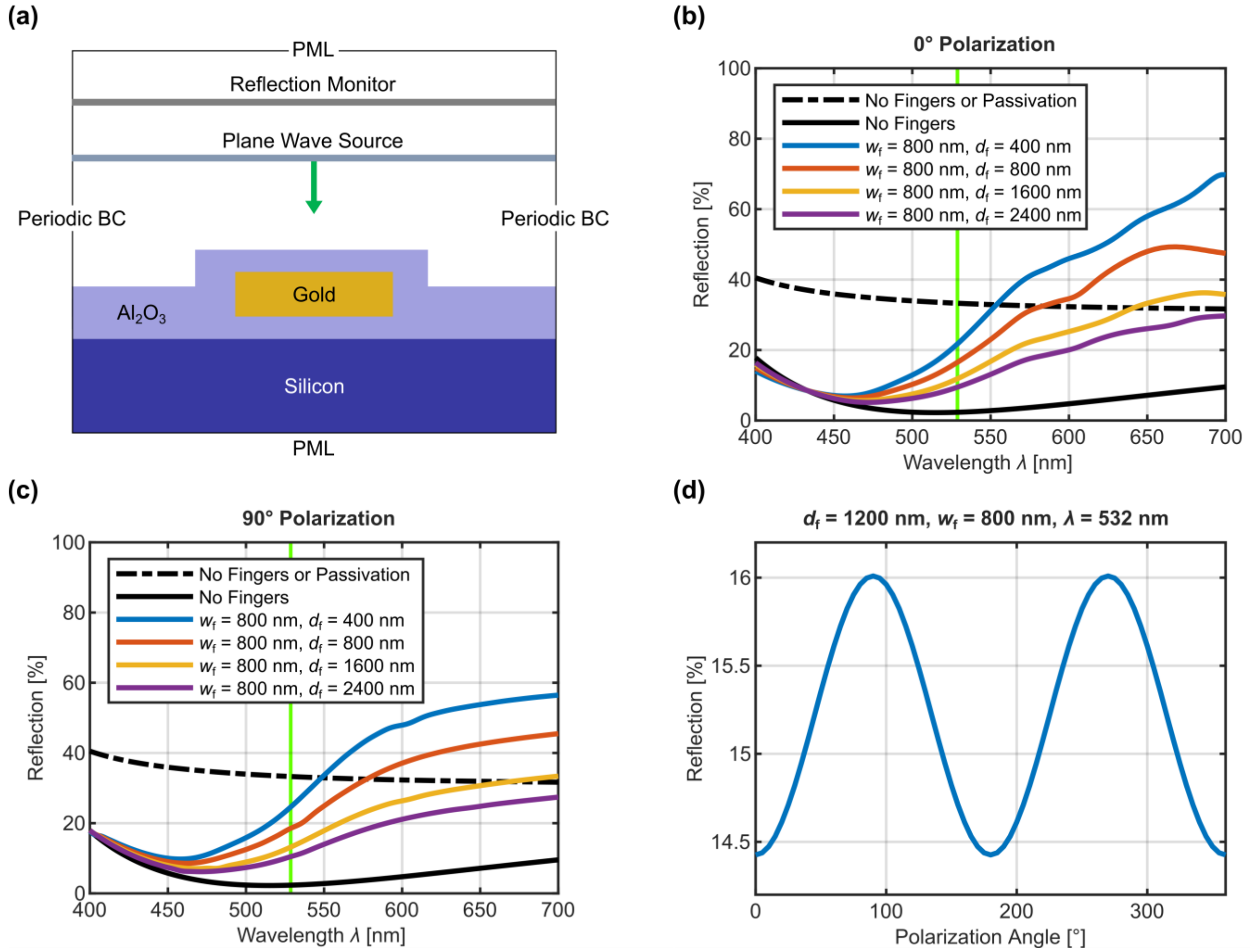

Figure S8. Reflection simulations of interdigitated finger structures

(a) Setup for the reflection simulations to estimate the reflectivity of various interdigitated finger structures. The 2D simulations consist of a silicon substrate, a 20 nm Al_2O_3 insulator and 60 nm thick gold finger. The 50 nm thick Al_2O_3 conformly follows the structure as expected for ALD-grown layers. For simplicity the graphene layer is omitted as the low absorption only marginally affect the simulations. To the left and right periodic boundary conditions (BC) and at the bottom and top perfectly matched layer (PML) BCs are used. A reflection monitor is placed above a plane wave source and measures the optical power reflected by the sample. Any portion of the incoming light, which is not reflected is either absorbed in the silicon substrate or lost due to ohmic losses in gold. (b) Reflection in dependence of the wavelength λ for various finger structure

configurations for a polarization angle of 0° (electric field perpendicular to the finger axis). While introducing the passivation has a clear antireflecting effect, the interdigitated fingers increase the reflections. (c) The same reflection measurements as shown in (b), but for a polarization angle of 90° (electric field parallel to the finger axis). (d) The reflection of a sample with an interdigitated finger structure as used for the measurement in Fig. 6 of the main text (d_f of 1200 nm and a finger width w_f of 800 nm) at a laser wavelength of 532 nm in dependence of the polarization angle (0°: E-field perpendicular to the finger axis). The reflection is only weakly polarization dependent.

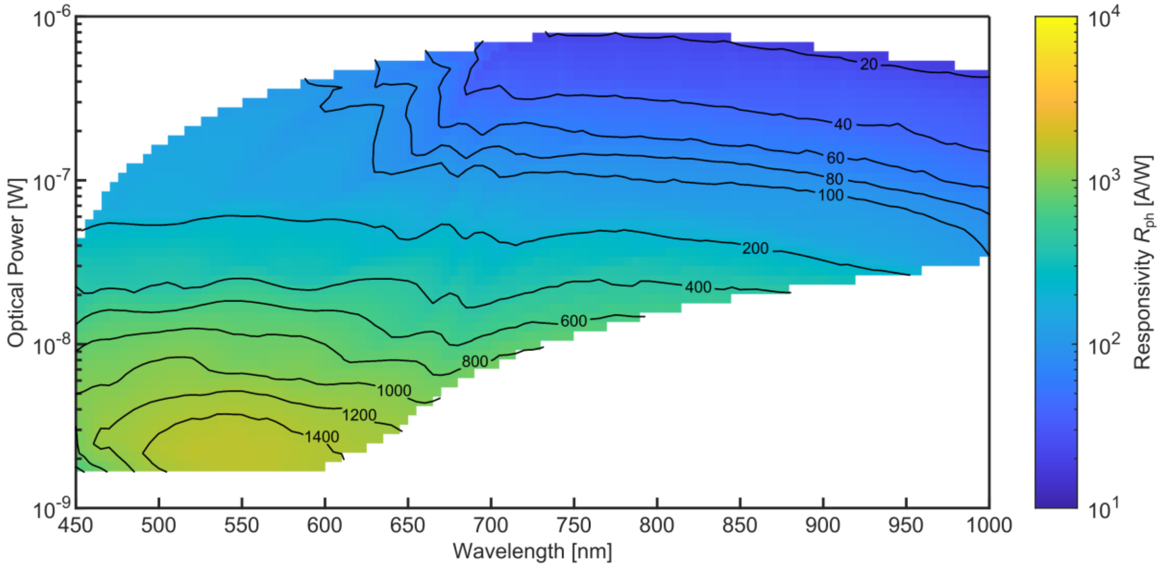

Figure S9. Spectral responsivity at different optical powers

Responsivity in dependence of the wavelength and incident optical power for an enhanced device with a semi-transparent top-gate and interdigitated fingers (w_f = 800 nm, d_f = 1200 nm). The laser spot size was comparable for all wavelengths. The measurement has been limited by the calibration measurements of the used neutral density filters at the lower end of optical powers.

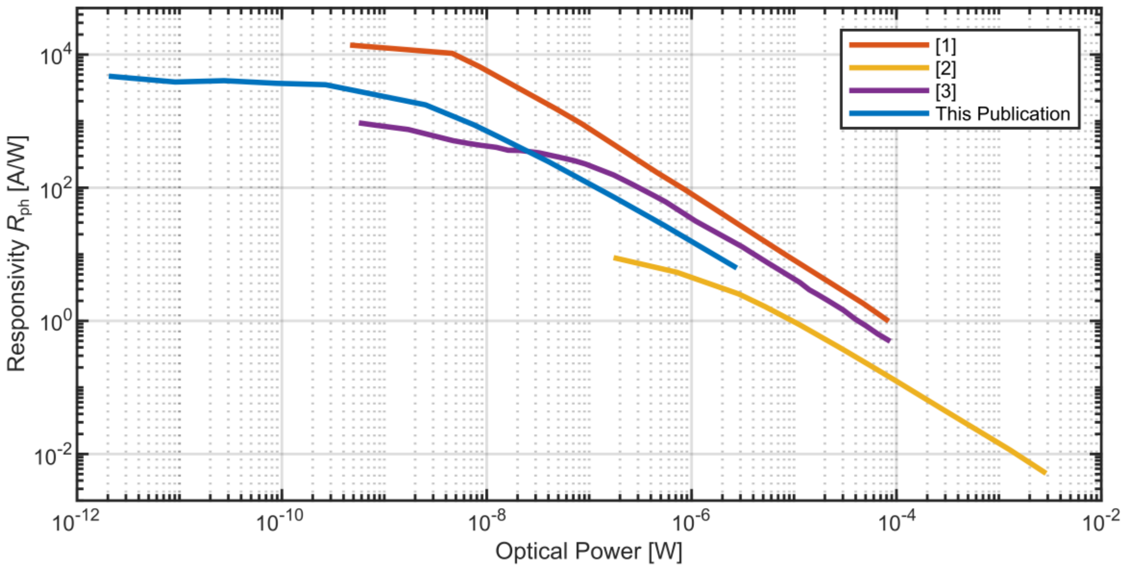

Figure S10. Comparison to demonstrations in literature

Responsivity at different optical powers for comparable literature demonstrations of interfacial photogating devices and our device. Due to the strong power-dependent responsivity only publications which include power sweep measurements have been considered. References: [1][4], [2][5], [3][6]. The device presented in this work shows the measured largest dynamic range as well as the lowest detected powers.

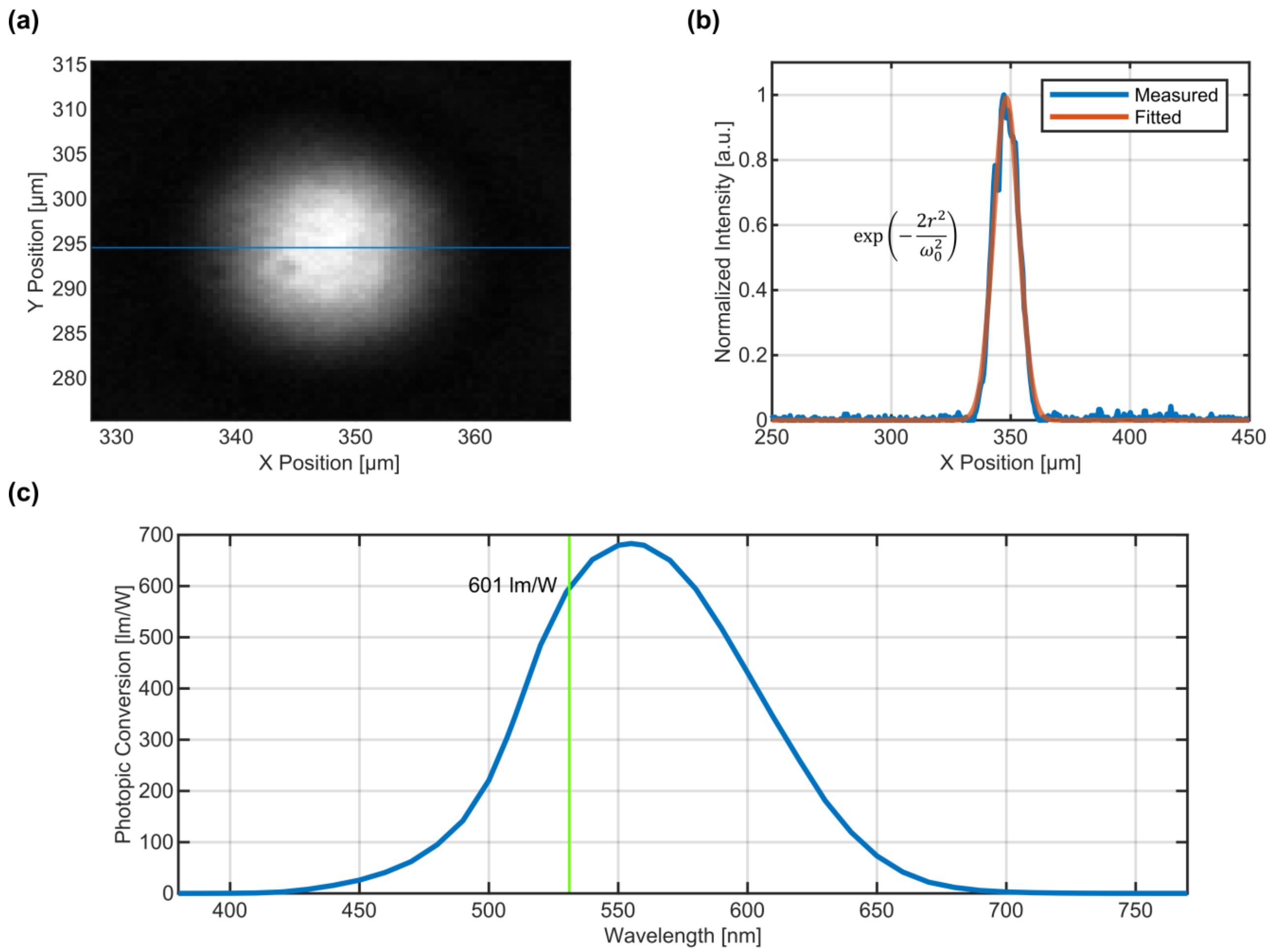

Figure S11. Photometric calculations

(a) Laser spot (532 nm laser) as used for the power sweep measurements in Figure 6 of the main text. (b) Cut-line from (a) together with a fitted Gaussian curve as shown in the equation. This fit provides a measure of the beam waist radius ω_0, which has a value of 10.6 μm. With this value the intensity of the Gaussian beam I_{opt} can be calculated as $I_{\mathrm{opt}} = P_{\mathrm{opt}}/(\pi\omega_0^2/2)$, where P_{opt} is the measured optical power of the laser beam. (c) The photopic conversion factor relates photometric with radiometric units. With help of the intensity calculation, the illuminance E_{v} can be calculated by taking into account the photopic conversion factor at 532 nm as follows $E_{\mathrm{v}} = 601(\mathrm{lm/W}) \cdot I_{\mathrm{opt}}$.

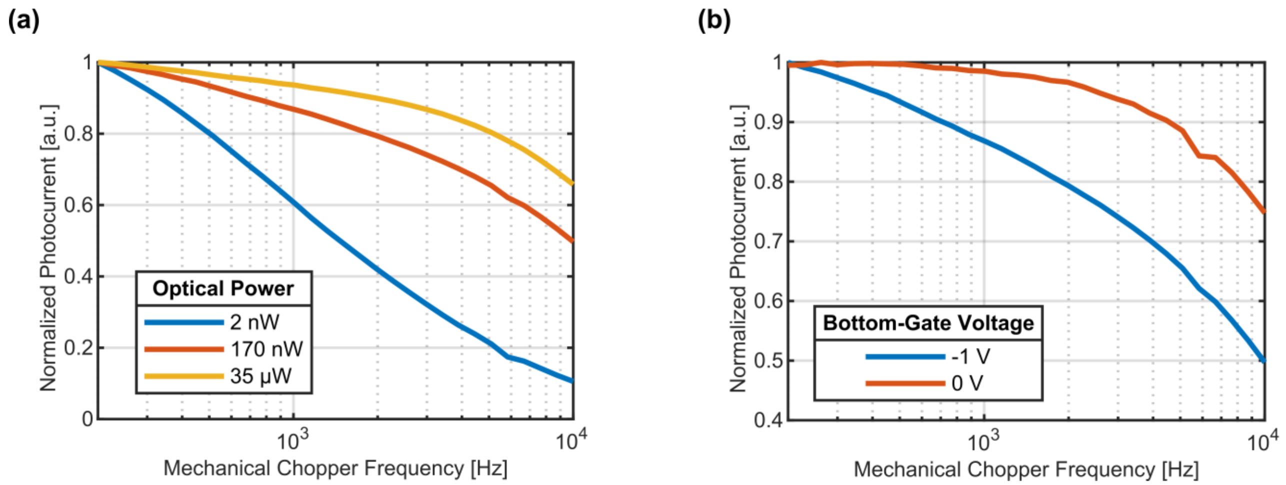

Figure S12. Power and bias dependence of frequency measurements

(a) Frequency sweep measurement of an enhanced device (1.2 μm finger-to-finger distance, 800 nm finger width, with a top-gate structure) as used for the frequency sweeps shown in Fig. 6 of the main text at different optical powers. The device is biased with 0.1 V_{SD}, -1 V V_{BG} and 1.5 V V_{TG}. At higher powers the device exhibits a faster response. This can be attributed to the fact that under depletion biasing (as is the case under -1 V V_{BG}) the increasing power levels lead to an increased amount of photogenerated charge carriers, which reduces the lifetime of the collected minority charges at the Si/Al_2O_3 interface. This reduction in lifetime leads to a faster photoresponse (b) Frequency sweep measurement of the same device under different bottom-gate voltage conditions. Under -1 V, with which silicon is biased into the depletion mode, the response time is longer and thus the photoresponse slower as compared to the case for 0 V bottom-gate voltage.

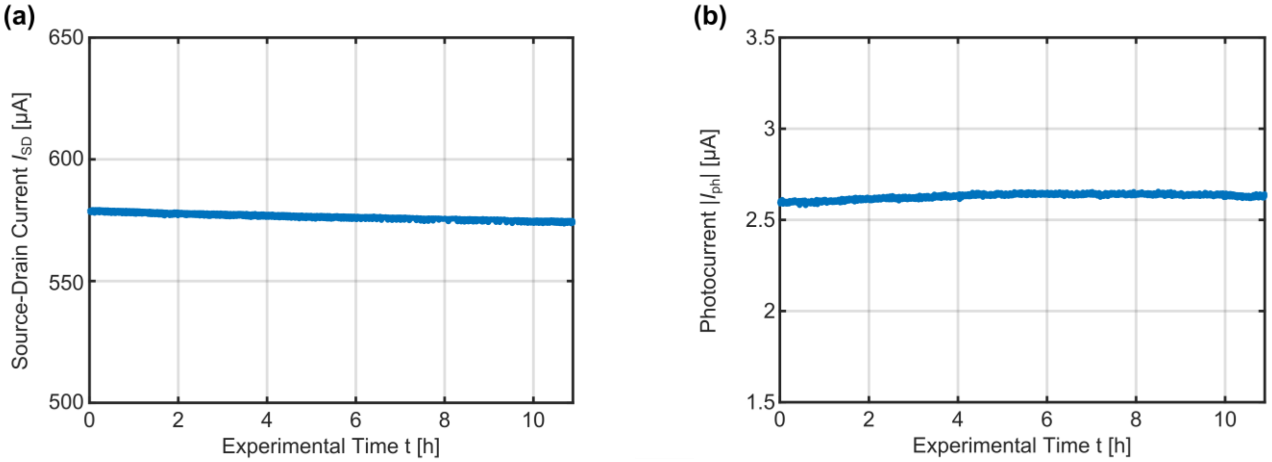

Figure S13. Stability measurement of the presented photodetectors

Measurement of the source-drain current and the photocurrent for a time span of more than 10 hours for an enhanced device with a semi-transparent top-gate and interdigitated fingers (w_f = 800 nm, d_f = 1200 nm). These stability measurements suggest that the device operates stably under ambient conditions in air and no performance degradation has been observed.

Reference	R_{ph} [A/W]	P_{min} [W]	τ_r [s]	λ [nm]	Channel Material	Gain Medium	Mechanism
**This work**	$4.7\cdot10^{3}$	$2\cdot10^{-12}$	$1.2\cdot10^{-3}$	532	CVD Graphene	Si	Interfacial
**4**	$1.4\cdot10^{4}$	$4.7\cdot10^{-11}$	$2\cdot10^{-6}$	532	CVD Graphene	Si	Interfacial
**5**	$4.58\cdot10^{1}$	$1\cdot10^{-7}$	$2\cdot10^{-5}$	633	CVD Graphene	Si	Interfacial
**6**	$1\cdot10^{3}$	$6\cdot10^{-10}$	$7.6\cdot10^{-7}$	514	ME Graphene	Si	Interfacial
**7**	$1\cdot10^{3}$	$1.4\cdot10^{-9}$	$1.2\cdot10^{-6}$	514	ME Graphene	Si	Interfacial
**8**	$8\cdot10^{2}$	$3\cdot10^{-4}$	$1\cdot10^{-5}$	632	ME Graphene Nanoribbons	Si	Interfacial
**9**	$1\cdot10^{3}$	$4\cdot10^{-11}$	$2.2\cdot10^{-6}$	520	ME Graphene	InGaAs	Interfacial
**10**	$8\cdot10^{3}$	$1\cdot10^{-9}$	$1.5\cdot10^{-1}$	500	SOI	CVD MoS2 & Si	Interfacial
**11**	$2.5\cdot10^{3}$	$1.8\cdot10^{-9}$	$6\cdot10^{-1}$	635	CVD Graphene	Si	D^2GOS
**12**	$1\cdot10^{4}$	$2\cdot10^{-9}$	$1\cdot10^{1}$	635	CVD Graphene	Si	D^2GOS
**13**	$6\cdot10^{4}$	$< 1\cdot10^{-9}$	$1\cdot10^{-6}$	532	CVD Graphene	Si	D^2GOS
**14**	$1\cdot10^{10}$	$2\cdot10^{-14}$	$2.73\cdot10^{-1}$	450	CVD Graphene	Cu2O QD	Direct
**15**	$1\cdot10^{7}$	$8\cdot10^{-15}$	$2\cdot10^{0}$	532	ME Graphene	PbS QD	Direct

Table S1. Table for comparing various photogating devices

The table shows various photodetectors utilizing the photogating effect. This table contains recent relevant works, but it is not complete. R_{ph} is the photoresponsivity, P_{min} the minimum measured power, τ_{r} the slowest time component of the photoresponse (rise or fall time) and λ the wavelength at which the values were measured. CVD stands for chemical vapor deposition, ME for mechanical exfoliation, SOI for silicon-on-insulator and QD for quantum dots. We differentiate between three photogating mechanisms, namely the interfacial, the deep-depletion graphene-oxide-semiconductor (D^2GOS) and the direct photogating mechanisms.

REFERENCES

(1) Lee, J. E.; Ahn, G.; Shim, J.; Lee, Y. S.; Ryu, S. Optical Separation of Mechanical Strain from Charge Doping in Graphene. *Nat. Commun.* **2012**, *3*, 1024. DOI: 10.1038/ncomms2022.

(2) Koepfli, S. M.; Baumann, M.; Koyaz, Y.; Gadola, R.; Güngör, A.; Keller, K.; Horst, Y.; Nashashibi, S.; Schwanninger, R.; Doderer, M.; et al. Metamaterial Graphene Photodetector with Bandwidth Exceeding 500 Gigahertz. *Science* **2023**, *380*, 1169-1174. DOI: 10.1126/science.adg8017.

(3) Mišeikis, V.; Marconi, S.; Giambra, M. A.; Montanaro, A.; Martini, L.; Fabbri, F.; Pezzini, S.; Piccinini, G.; Forti, S.; Terrés, B.; et al. Ultrafast, Zero-Bias, Graphene Photodetectors with Polymeric Gate Dielectric on Passive Photonic Waveguides. *ACS Nano* **2020**, *14*, 11190-11204. DOI: 10.1021/ACSNANO.0C02738.

(4) Tao, L.; Li, H.; Sun, M.; Xie, D.; Li, X.; Xu, J.-B. Enhanced Photoresponse in Interfacial Gated Graphene Phototransistor with Ultrathin Al_2O_3 Dielectric. *IEEE Electron Device Lett.* **2018**, *39*, 987-990. DOI: 10.1109/LED.2018.2843804.

(5) Huang, Z.; Liu, J.; Zhang, T.; Jin, Y.; Wang, J.; Fan, S.; Li, Q. Interfacial Gated Graphene Photodetector with Broadband Response. *ACS Appl. Mater. Interfaces* **2021**, *13*, 22796-22805. DOI: 10.1021/acsami.1c02738.

(6) Guo, X.; Wang, W.; Nan, H.; Yu, Y.; Jiang, J.; Zhao, W.; Li, J.; Zafar, Z.; Xiang, N.; Ni, Z.; et al. High-Performance Graphene Photodetector Using Interfacial Gating. *Optica* **2016**, *3*, 1066-1070. DOI: 10.1364/OPTICA.3.001066.

(7) Wang, W.-H.; Du, R.-X.; Guo, X.-T.; Jiang, J.; Zhao, W.-W.; Ni, Z.-H.; Wang, X.-R.; You, Y.-M.; Ni, Z.-H. Interfacial Amplification for Graphene-Based Position-Sensitive-Detectors. *Light: Sci. Appl.* **2017**, *6*, e17113. DOI: 10.1038/lsa.2017.113.

(8) Yu, J.; Zhong, J.; Kuang, X.; Zeng, C.; Cao, L.; Liu, Y.; Liu, Z. Dynamic Control of High-Range Photoresponsivity in a Graphene Nanoribbon Photodetector. *Nanoscale Res. Lett.* **2020**, *15*, 124. DOI: 10.1186/s11671-020-03352-7.

(9) Cao, G.; Wang, F.; Peng, M.; Shao, X.; Yang, B.; Hu, W.; Li, X.; Chen, J.; Shan, Y.; Wu, P.; et al. Multicolor Broadband and Fast Photodetector Based on InGaAs–Insulator–Graphene Hybrid Heterostructure. *Adv. Electron. Mater.* **2020**, *6*, 1901007. DOI: 10.1002/aelm.201901007.

(10) Deng, J.; Zong, L.; Zhu, M.; Liao, F.; Xie, Y.; Guo, Z.; Liu, J.; Lu, B.; Wang, J.; Hu, W.; et al. MoS_2/HfO_2/Silicon-On-Insulator Dual-Photogating Transistor with Ambipolar Photoresponsivity for High-Resolution Light Wavelength Detection. *Adv. Electron. Mater.* **2019**, *29*, 1906242. DOI: 10.1002/adfm.201906242.

(11) Howell, S. W.; Ruiz, I.; Davids, P. S.; Harrison, R. K.; Smith, S. W.; Goldflam, M. D.; Martin, J. B.; Martinez, N. J.; Beechem, T. E. Graphene-Insulator-Semiconductor Junction for Hybrid Photodetection Modalities. *Sci. Rep.* **2017**, *7*, 14651. DOI: 10.1038/s41598-017-14934-4.

(12) Ruiz, I.; Beechem, T. E.; Smith, S.; Dickens, P.; Paisley, E. A.; Shank, J.; Howell, S. W.; Sarma, R.; Draper, B. L.; Goldflam, M. D. Interface Defect Engineering for Improved Graphene-Oxide-Semiconductor Junction Photodetectors. *ACS Appl. Nano Mater.* **2019**, *2*, 6162-6168. DOI: 10.1021/acsanm.9b00978.

(13) Liu, W.; Lv, J.; Peng, L.; Guo, H.; Liu, C.; Liu, Y.; Li, W.; Li, L.; Liu, L.; Wang, P.; et al. Graphene Charge-Injection Photodetectors. *Nat. Electron.* **2022**, *5*, 281-288. DOI: 10.1038/s41928-022-00755-5.

(14) Liu, Q.; Tian, H.; Li, J.; Hu, A.; He, X.; Sui, M.; Guo, X. Hybrid Graphene/Cu_2O Quantum Dot Photodetectors with Ultrahigh Responsivity. *Adv. Opt. Mater.* **2019**, *7*, 1900455. DOI: 10.1002/adom.201900455.

(15) Konstantatos, G.; Badioli, M.; Gaudreau, L.; Osmond, J.; Bernechea, M.; de Arquer, F. P. G.; Gatti, F.; Koppens, F. H. L. Hybrid Graphene–Quantum Dot Phototransistors with Ultrahigh Gain. *Nat. Nanotechnol.* **2012**, *7*, 363-368. DOI: 10.1038/nnano.2012.60.
